# Impact of the Renin–Angiotensin System on the Endothelium in Vascular Dementia: Unresolved Issues and Future Perspectives

**DOI:** 10.3390/ijms21124268

**Published:** 2020-06-16

**Authors:** Fatima Y. Noureddine, Raffaele Altara, Fan Fan, Andriy Yabluchanskiy, George W. Booz, Fouad A. Zouein

**Affiliations:** 1 Department of Pharmacology and Toxicology, Faculty of Medicine, American University of Beirut, Beirut 1107 2020, Lebanon; nouriddinef@gmail.com; 2 Institute for Experimental Medical Research, Oslo University Hospital and University of Oslo, and KG Jebsen Center for Cardiac Research, 0424 Oslo, Norway; raffaele.altara@medisin.uio.no; 3 Department of Pharmacology and Toxicology, School of Medicine, The University of Mississippi Medical Center, Jackson, MS 39216, USA; ffan@umc.edu (F.F.); gbooz@umc.edu (G.W.B.); 4 Center for Geroscience and Healthy Brain Aging, University of Oklahoma Health Sciences Center, Oklahoma City, OK 73104, USA; andriy.yab@gmail.com

**Keywords:** blood–brain barrier, ACE1, ACE2, AT2 receptor, Mas receptor, Ang(1–7)

## Abstract

The effects of the renin–angiotensin system (RAS) surpass the renal and cardiovascular systems to encompass other body tissues and organs, including the brain. Angiotensin II (Ang II), the most potent mediator of RAS in the brain, contributes to vascular dementia via different mechanisms, including neuronal homeostasis disruption, vascular remodeling, and endothelial dysfunction caused by increased inflammation and oxidative stress. Other RAS components of emerging significance at the level of the blood–brain barrier include angiotensin-converting enzyme 2 (ACE2), Ang(1–7), and the AT2, Mas, and AT4 receptors. The various angiotensin hormones perform complex actions on brain endothelial cells and pericytes through specific receptors that have either detrimental or beneficial actions. Increasing evidence indicates that the ACE2/Ang(1–7)/Mas axis constitutes a protective arm of RAS on the blood–brain barrier. This review provides an update of studies assessing the different effects of angiotensins on cerebral endothelial cells. The involved signaling pathways are presented and help highlight the potential pharmacological targets for the management of cognitive and behavioral dysfunctions associated with vascular dementia.

## 1. Introduction

According to the National Institute on Aging (https://www.nia.nih.gov/health/vascular-contributions-cognitive-impairment-and-dementia), vascular contributions to cognitive impairment and dementia (VCID) result from injuries or pathologies of blood vessels that supply the brain and lead to a significant decline in cognitive function and memory. The size, location, and number of injuries correspond to the severity of the dysfunction. VCID encompasses at least seven forms of dementia, including (1) vascular dementia, which corresponds to cerebrovascular injury or disorder that causes gradual decline in memory and cognition, and shares some symptoms with Alzheimer’s disease; (2) vascular cognitive impairment, which is caused by vascular or brain pathologies, and corresponds to alterations in memory, attention, language, and reasoning ability that are not as significant as to distort daily performance; (3) post-stroke dementia, which is very likely to develop months after a major stroke; and (4) multi-infarct dementia, which develops as a result of a number of mini-strokes and, more potentially, small strokes (infarcts); the risk of dementia increases in a bilateral stroke, and the impaired function depends upon the affected area. Other forms of VCID are cerebral autosomal dominant arteriopathy with subcortical infarcts and leukoencephalopathy (CADASIL), subcortical vascular dementia, and cerebral amyloid angiopathy. Vascular dementia (VaD) is the second most common form of dementia after Alzheimer’s disease, contributing to nearly 17% of all dementias [1,2]. The risk of VaD increases with age such that it doubles approximately every five years [1]. 

The blood–brain barrier (BBB) is a protective border that supports selective exchange between circulating blood and the extracellular fluid of the central nervous system. The barrier properties of the endothelial cells comprising the BBB depend on the expression of tight junction proteins between adjacent cells. The BBB is considered part of the neurovascular unit (NVU) whose function is to couple cerebral blood flow to neuronal demands (neurovascular coupling) [3]. The NVU is also composed of pericytes, basement membranes, and astrocyte end-feet processes. The disruption of either BBB or neurovascular coupling responses contributes to cognitive dysfunction and other pathologies associated with Alzheimer’s disease [4]. This has been shown to play a key role in the hypertension-induced enhancement of cognitive dysfunction [5]. In addition, hypertension can induce cerebral artery remodeling and alter endothelium-dependent vascular responses, thus impacting blood flow to the brain [6,7]. The relationship between midlife hypertension and decreased cerebral blood flow was demonstrated in an Alzheimer’s disease mouse model [8]. Midlife patients with atrial fibrillation also showed an important association between hypertension burden and dementia risk [9]. Interestingly, Alzheimer’s disease mice also showed increased amyloid levels in cerebral vessels and brain tissue [10]. Similar vascular-related mechanisms contribute to the increased risk of dementia associated with traumatic brain injury and several age-related conditions like myocardial infarction and ischemic stroke [11,12,13]. 

## 2. Role of the Renin–Angiotensin System 

**Figure 1:**
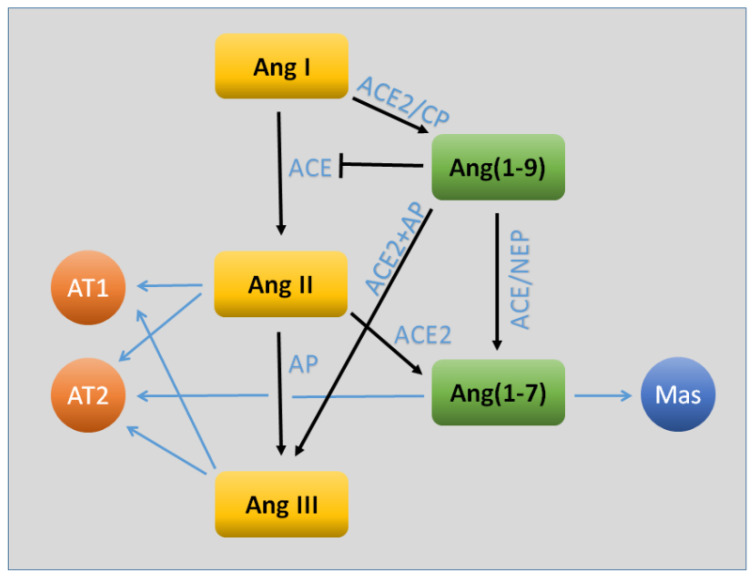
Key components of the angiotensin (Ang) system relevant to the blood–brain barrier. The traditional arm of the system (shown in yellow) consists of the sequential processing of Ang I, Ang II, and Ang III. The latter two act upon the AT1 receptor to cause a number of physiological actions, including (for the most part) vasoconstriction, increased oxidative stress, and inflammation. The AT2 receptor may contribute as well to oxidative and inflammatory signaling, but may oppose vasoconstriction. In recent years, evidence has been found for a parallel arm (shown in green) that largely opposes the traditional actions of Ang II/Ang III. The primary effector of this system is Ang(1–7), which acts through the Mas receptor. Both Ang(1–9) and Ang(1–7) may also act through AT2R. Abbreviations: ACE, angiotensin-converting enzyme; ACE2, angiotensin-converting enzyme 2; AP, aminopeptidase; CP, carboxypeptidase; NEP, neutral endopeptidase.

The renin–angiotensin–aldosterone system (RAS) plays a key role in different physiological functions, mainly ones associated with the cardiovascular system, including the modulation of vascular tone, fluid volume, cardiac output, vascular wall integrity, and cellular growth [14]. RAS can also be involved in the pathophysiology of several diseases like hypertension, atherosclerosis, and chronic kidney diseases [15]. Angiotensin II (Ang II) is the main bioactive product of the RAS system (Figure 1) and acts mainly through activation of the Ang II type 1 receptor (AT1R) and its downstream signaling cascade [15]. Other RAS components include Ang(1–7), known to counteract Ang II/AT1R through its Mas receptor (MasR), Ang(2–8), Ang(3–8), Ang IV, and Ang(1–12) [15]. In the brain, Ang II-mediated AT1R activation is associated with elevated neuronal oxidative stress and inflammation and contributes to cognitive dysfunction [15]. It is now established that the brain possesses its own local RAS, a fuller description of which can be found elsewhere [15].

Circulating Ang II can also impact the brain through the cerebral microvasculature by modulating vascular tone and promoting vascular hypertrophy and fibrosis [15]. Pharmacological and genetic approaches have shown that Ang II may cause either vasodilation or vasoconstriction via AT1R activation of cerebral endothelial cells (CECs). In normotensive pigs and rats, for instance, AT1R activation on endothelial cells of cerebral arteries and arterioles leads to vasodilation likely via a cyclooxygenase-derived factor [16,17]. In contrast, Ang II-induced reactive oxygen species in the endothelium via AT1R decrease nitric oxide (NO) bioavailability and promote endothelial dysfunction and vascular tone impairment [15]. For example, Ang II-induced AT1R activity increases the vasoconstriction of middle cerebral arteries isolated from the elastin haploinsufficient (Eln^+/–^) mouse model of aortic stiffness. IL-1β gene expression was significantly higher in Eln^+/–^ mice compared to wild type (WT) Eln^+/+^ suggesting an enhanced inflammatory response in this model [18].

## 3. Endothelium

**Figure 2:**
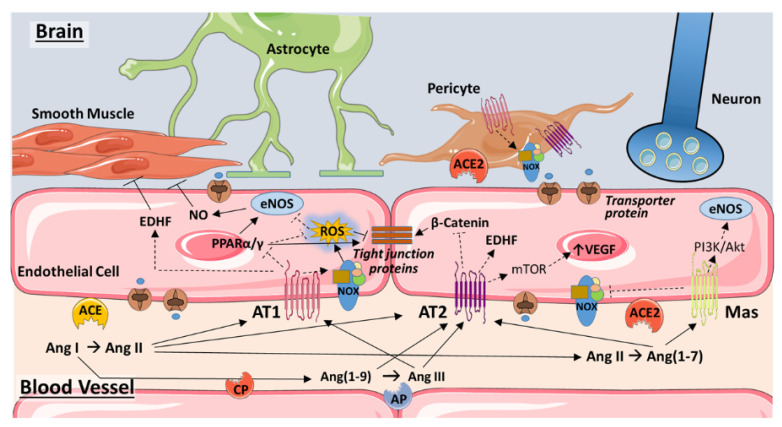
Angiotensin signaling in the endothelium of the blood–brain barrier. Traditional and novel components of the renin–angiotensin system (RAS) converge at the level of the endothelium to modulate blood flow, vascular remodeling, and brain permeability. Ang II formed by the actions of ACE on Ang I may act on the AT1 receptor to induce either vasodilation, via production of EDHF, or vasoconstriction by stimulating ROS production via Nox2 and attenuating eNOS-induced NO production. Inhibition of PPARα/γ may exacerbate ROS production. Ang II-induced ROS and inhibition of PPARα/γ may act synergistically to increase blood–brain barrier permeability by impeding the expression or functioning of tight junction proteins. ROS contributes as well to adverse vascular remodeling and vessel rarefaction that impedes blood flow and occurs with hypertension and aging. Through AT2R, Ang II may induce vasodilation via EDHF production. AT2R is also linked to cardioprotective effects via vascular endothelial growth factor (VEGF) expression, as well as strengthening of interendothelial junctions via inhibition of β-catenin. However, VEGF is also implicated in the disruption of the blood–brain barrier. NO may also increase AT2R expression, which in turn may reduce ACE levels (not shown). Ang(1–9), Ang(1–7), and Ang III may activate AT2R as well, with Ang III also acting on AT1R. Ang(1–7), formed from Ang II by ACE2, acts upon the Mas receptor to constitute a protective arm of RAS through inhibition of Nox2 and activation of eNOS. In pericytes, AT1R activation may induce oxidative stress by increasing Nox4, thereby triggering pericyte loss and compromising the blood–brain barrier. Based on recent research in the heart, brain pericytes may also express ACE2. Not shown are the RAS components associated with astrocytes and neurons. See text for additional details. Abbreviations: ACE, angiotensin converting enzyme; AP, aminopeptidase; AT1, angiotensin II type 1 receptor; AT2, angiotensin II type 2 receptor; CP, carboxypeptidase; EDHF, endothelium-derived hyperpolarizing factor; eNOS, endothelial nitric oxide synthase; mTOR, mammalian target of rapamycin; NO, nitric oxide; NOX, NADPH oxidase; PI3K, phosphoinositide 3-kinase; PPARα/γ, peroxisome proliferator-activated receptor alpha/gamma; ROS, reactive oxygen species; VEGF, vascular endothelial growth factor. Some images are from Servier Medical Art (https://smart.servier.com/).

CECs are key constituents of the blood–brain barrier (Figure 2) and are of upmost importance in the functions of the neurovascular unit (NVU) [19]. Blood–brain barrier impairment is a sign of cognitive dysfunction in humans [20] and seems to be in direct association with vascular dementia and early stages of dementia in Alzheimer’s disease [19,21]. CEC injury perturbs CEC–neural cell interactions through neuronal inflammation and increased ROS production and apoptosis, causing therefore astrogliosis, activation of microglia, disrupted synaptic plasticity, compromised neuronal axons, and white matter damage, leading subsequently to an impaired neurological and cognitive function [19,22]. In a Tgαq*44 mouse model of slowly developing heart failure, inflammation of the cortical endothelium and impaired NO-dependent endothelial function in cortical arterioles contributed to the development of cognitive dysfunction [23]. In the same context, blockade of miR-133a, a contributor to diabetes-induced cerebral endothelial dysfunction, improved learning abilities and memory in a streptozotocin-induced diabetes rat model [24]. Unsurprisingly, numerous studies link the above-mentioned effects to the RAS system and its downstream counterparts and signaling cascades, as detailed in the subsections below.

### 3.1. Angiotensin and AT1R

Accumulating evidence suggests that blood–brain barrier impairment is associated with vascular dementia with a direct link to Ang II effects. Ang II stimulates ROS production, increases inflammation, impacts neurovascular coupling, promotes vascular remodeling, and disrupts vasomotor function [25]. In this context, Ang II was shown to increase the permeability of primary human coronary artery endothelial cell layers through distorting the expression and distribution of zona occludens-1 (ZO-1), a protein associated with the tight junctions and a key player in the barrier properties of the endothelium of cerebral blood vessels [26]. In contrast to this finding, astrocyte-derived Ang II was reported to be necessary for blood–brain barrier maintenance by AT1R-mediated phosphorylation of the tight junction protein occludin and its translocation to lipid raft membrane microdomains in CECs [27]. These disparate findings hint at the possible opposing roles of the circulating vs. local levels of Ang II in the disruption and maintenance of the blood–brain barrier, respectively. 

In another study conducted with an Ang II-induced model of hypertension, Ang II altered the bradykinin-dependent biphasic response of isolated basilar artery specimens and diminished the associated amounts of NO, suggesting that Ang II-induced hypertension impairs the function of cerebrovascular endothelium [28]. Disrupted amounts and signaling of NO is a hallmark of oxidative stress involvement and Ang II is well known to increase cerebrovascular ROS generation [25]. Multiple studies highlight the role of Ang II-induced dysfunction of the cerebral microvascular endothelium via increased superoxide production through its activation of the AT1R [29,30]. Specifically, Ang II-AT1R induced oxidative stress is mediated through Nox2-NADPH oxidase dependent activity [31,32]. The upregulation of AT1R in human umbilical vein endothelial cells exposed to particulate matter (PM) 2.5 was accompanied by an increase in ROS production [33]. The same cell model cultured in the presence of Ang II showed increased oxidative stress levels accompanied with increased NADPH oxidase activity and Nox2 expression [34]. 

Nox2-induced oxidative stress is suggested to be an early characteristic of aging-related cerebrovascular dysfunction, and aging Nox2 knockout (KO) mice were shown to exhibit less neuronal and vascular oxidative stress and DNA damage than matched aging WT mice [32,35]. The contribution of Nox2 to human brain aging was further demonstrated by examining post-mortem midbrain tissues of young and elderly adults, which proved an age-related increase in Nox2-dependent ROS levels [32]. Addressing signaling mechanisms, recent evidence suggests that some of the Ang II-AT1R effects in brain endothelial cells might be attributed to regulating the ligand-activated transcription factors, peroxisome proliferator-activated receptor α (PPARα) and PPARγ [36,37]. Treating human brain microvascular endothelial cell cultures with Ang II enhanced their permeability through both para- and trans-cellular pathways, impaired the expression and arrangement of several junction and transport proteins, and deactivated PPARα signaling. The aforementioned effects were reversed when PPARα agonists were added [36]. PPARα can modulate endothelial function by the attenuation of endothelin-1 (ET-1), which is involved in several cerebral pathological complications [38]. In piglet cerebral microvascular endothelial cells, PPARα activation reduced basal and vasoactive agent-induced increase in ET-1 levels. This effect was mediated through the upregulation of eNOS, elevation of NO, and regulation of PKC signaling pathways [38]. PPARγ, on the other hand, holds antioxidant and anti-inflammatory effects in endothelium [37] and has been shown to counteract Ang II-induced endothelial dysfunction [39]. For instance, overexpression of an adipokine, CTRP6 in spontaneously hypertensive rats (SHRs) decreased Ang II expression, alleviated Ang II-induced hypertension and endothelial dysfunction, and increased p-PPARγ and p-ERK1/2 protein levels in the brain, suggesting that the protective effects of CTRP6 are mediated through ERK signaling and PPARγ activation [39]. 

Infusing PPARγ mutant transgenic mice with Ang II attenuated isolated carotid artery response to acetylcholine, increased superoxide levels, and upregulated the pro-oxidant Nox2, while downregulating anti-oxidant genes expression [37]. Similarly, in response to high-fat diet, basilar arteries of transgenic mice expressing endothelium-selective dominant-negative mutants of PPARγ exhibited altered acetylcholine-induced dilation compared to that observed in WT mice [40]. Basilar artery dilation was corrected after superoxide scavenger treatment. Subsequent analysis of the expression of genes in the oxidative stress pathway confirmed ROS involvement. Transgenic mice on high-fat diet were also more sensitive to an Ang II pressor response compared to WT mice [40]. Similar findings were recorded with PPARγ endothelium-selective dominant-negative mutations in mice on low-salt diet known to stimulate renin and Ang II activation. The observed dysfunctions due to PPARγ loss were restored once transgenic mice received AT1R blockers, providing evidence that AT1R contributes to the endothelial dysfunction induced by PPARγ inhibition [41]. 

Therapeutically, AT1Rs represent an important target in different peripheral and, recently, brain disorders. The neuroprotective effects of AT1R blockers (ARBs) were observed in different in vivo disease models, including that of traumatic brain injury, stroke, dementia, and Alzheimer’s disease [42]. ARBs can directly impact neurons, astrocytes, microglia, and endothelial cerebrovascular cells. In endothelial cells, ARBs have been shown to counteract Ang II-induced pathological vasoconstriction and attenuate several pro-inflammatory signaling pathways and ROS production [42]. Treating murine brain endothelial cells (mBECs) with ARBs, such as azilsartan, protects against tert-butyl hydroperoxide-induced cell injury; azilsartan reduced oxidative stress, mitochondrial dysfunction, apoptosis, and inflammatory response. Adding a PPARγ antagonist lowered cell viability suggestive of PPARγ involvement [43]. Several ARBs have been shown to activate PPARγ, those include telmisartan, candesartan, and losartan. Whether PPARγ activation and subsequent brain and vascular protective effects are independent of AT1R blockade remains unclear [42]. 

The mineralocorticoid receptor (MR) is another major key player in Ang II-induced vascular injury. MR and its ligand, aldosterone, are well known to regulate the sodium and potassium balance in the kidneys [44]. Recently, MR has been recognized as a functional hormone-activated transcription factor in vascular cells, including endothelial cells [44]. In vascular tissue, MR is thought to be activated mainly by the mineralocorticoid, aldosterone [44]. However, some of the Ang II/AT1R cardiovascular effects appear to be mediated through MR [45]. Strong evidence supports a crosstalk between MR and AT1R involving unknown mechanisms [44]. Inhibition of MR in Ang II-induced hypertensive mice prevented the altered endothelium-dependent dilation and increased myogenic tone of cerebral parenchymal arterioles. MR antagonists upregulate angiotensin-converting enzyme 2 (ACE2), a constituent of the protective arm of the RAS [46]. MR antagonists also reduced cortical microglia density and improved hypertension-induced cognitive function [47]. Furthermore, temporary MR inhibition improved endothelium-dependent dilation and reduced outer and lumen diameters of the middle cerebral artery in spontaneously hypertensive rats weeks after MR antagonist withdrawal, suggestive of MR involvement in hypertension-induced cerebral vascular remodeling [48]. 

### 3.2. ACE2

Arterial and venous endothelial cells express angiotensin-converting enzyme 2 (ACE2), which is now suggested to protect against ischemia-induced cerebral injuries. When exosomes of endothelial progenitor cells are transfected with lentivirus containing human ACE2 cDNA (ACE2-EPC-EXs), a protective effect on hypoxia/reoxygenation (H/R)-induced injury in cultured aging brain endothelial cells emerges [49]. However, only a partial protection is observed with ACE2-EPC-EXs ^anti-miR-18a^, suggestive of miR-18a involvement. Subsequent analysis revealed that the observed protection is mediated through anti-apoptotic and anti-oxidative effects via the miR-18a/Nox2/ROS pathway [49]. In a more recent study, ACE2-EPC-exosomes were shown to protect cerebral microvascular endothelial cells with Ang II-induced injury against apoptosis, ROS generation, mitochondrion fragmentation, and reduced tube formation and migration abilities. This effect is in part attributed to Ang II/Ang(1–7) imbalance with an overall increase in Ang(1–7) levels through ACE2 mediated Ang II hydrolysis [50]. 

The protective effects of ACE2 against age-induced oxidative stress and endothelial dysfunction is highlighted in several studies. Old-aged ACE2 KO mice showed worsened impairment of acetylcholine-induced cerebral artery vasodilation than that observed in old WT mice. Incubating cerebral arteries with a superoxide scavenger prior to ACh treatment rescued the endothelial function in ACE2 KO mice, indicating a direct a role of oxidative stress in the cerebrovascular dysfunction in association with ACE2 deficiency and aging [51]. Multiple lines of evidence confirmed that oxidative stress is a key mediator of ACE2 deficiency-induced cerebrovascular dysfunction. 

The overexpression of ACE2 in dysfunctional bone marrow endothelial progenitor cells from renin and angiotensinogen double transgenic (RA) mice elevated eNOS expression and NO production, while decreasing Nox2 and Nox4 expression and subsequent ROS production. This was accompanied by an enhancement of EPC migration and tube formation abilities. The ACE2 overexpression protective effects were countered by ACE2 or eNOS inhibitors, but enhanced with a Nox inhibitor [52]. The protective impact of neuronal ACE2 in the brain is believed to be mediated via similar mechanisms. ACE2 overexpression in the brain attenuated deoxycorticosterone acetate (DOCA)-salt-induced oxidative stress, amplified antioxidant enzyme activities, and lessened neuroinflammation by limiting the increase in COX-1 and COX-2 expression in the hypothalamic paraventricular nucleus [53]. In ischemic injury, neuronal ACE2 protective mechanisms were associated with the regulation of oxidative stress pathways. Renin–angiotensin double transgenic mice overexpressing cerebral ACE2 showed increased eNOS/NO expression and decreased Nox/ROS levels, and these were associated with lower basal mean arterial pressure, reduced middle cerebral artery occlusion-induced infarct volume, elevated cerebral blood flow, and enhanced neurological function and cerebral microvascular density in the pre-infarct area. The ability of MasR antagonism to reverse the ACE2 protective effects, along with the observed Ang(1–7)/AngII ratio increase in the cerebral parenchyma, suggests that the protective effects of ACE2 involve Ang(1–7)/MasR activation [54].

### 3.3. Ang(1–7) and the Mas Receptor

Ang(1–7) increases oxygen delivery to the brain through the cerebral microvessels via two main mechanisms: (1) stimulating angiogenesis and (2) enhancing blood flow via the upregulation of NO production [55,56,57]. Ang(1–7) treatment repressed pro-apoptotic activity, decreased oxidative stress, and upregulated NO formation in human cerebral microvascular endothelial cells otherwise seen with Ang II-induced dysfunction [56]. This was accompanied by a decrease in Nox2 expression and activation of the PI3K/Akt/eNOS pathway [56]. The Ang(1–7) above-mentioned protective effects, however, were blocked with MasR antagonists [56]. In another study, infusion with Ang(1–7) promoted brain angiogenesis in rats. Enriched capillary density and endothelial cell proliferation were associated with increased brain NO and VEGF expression and eNOS activity. Ang(1–7) also enhanced cerebral blood flow and reduced infarct volume and neurological deficits after permanent middle cerebral artery occlusion. The aforementioned effects were reversed by the action of a MasR antagonist and eNOS inhibitor, indicating that the Ang(1–7) protective effects are potentially mediated through a Mas/eNOS-dependent mechanism [57]. MasR is a G-protein-coupled receptor for the agonist Ang(1–7), widely expressed in neurons, microglia and cerebral vascular endothelium, and known to enhance cerebral blood flow while decreasing inflammation and ROS production [55]. 

In human umbilical vein endothelial cells cultured in the presence of both Ang II and increasing amounts of O-linked glycopeptide PNA5, a glucoside derivative of Ang(1–7) and agonist of MasR, ROS production decreased in a dose-dependent manner [55]. The observed PNA5 effects, however, were prevented in the presence of the MasR antagonist, A779 [55]. In the same study, a mouse model of vascular contribution to cognitive impairment and dementia/heart failure (VCID/HF) receiving a three-week daily PNA5 treatment showed diminished microglia/macrophage activation in the cortical meningeal region of the brain and decreased levels of inflammatory cytokines in mice sera [55]. Additional findings suggested that MasR-induced cerebral blood flow improvement and subsequent restoration of cognitive function are mediated mainly through the PI3K/Akt/eNOS pathway [55].

### 3.4. Other Novel RAS Components: AT2 and AT4 Receptors

In contrast to the damaging effects of excessive and sustained activation of AT1R by Ang II, AT2R activation is reported to exhibit anti-inflammatory and neuroprotective effects in vascular and CNS pathologies and has emerged as a potential therapeutic strategy to counterbalance AT1R [58,59]. AT2R-knockout mice subjected to transient bilateral carotid artery occlusion tended to have higher numbers of platelets rolling and adhering to endothelial cells in the pial vein and artery. This was attributed to the preferable action of Ang II on AT1R in the absence of AT2R activity [60]. On the other hand, when AT1R is blocked and AT2R stimulated, the junctions of human brain microvascular endothelial cells remained intact in response to ruptured Plasmodium falciparum-infected red blood cells as seen in cerebral malaria [61]. AT2R protective effects were attributed to inhibition of β-catenin and strengthening of interendothelial junctions. Moreover, modulation of Ang II receptors had protective effects in a mouse cerebral malaria model as evidenced by reduced cerebral hemorrhages and increased survival, whereas AT2R-deficient mice were found to be more susceptible to cerebral malaria. Similarly, spontaneously hypertensive rats prone to middle cerebral artery occlusion (MCAO) and reperfusion showed decreased cognitive dysfunction, Aβ_1–42_ cellular accumulation, and chronic-reactive microgliosis in brain tissue in the presence of continuous administration of AT2R agonist and AT1R blocker [62]. When treated with AT2R agonist at the time of reperfusion, MCAO rats showed preserved neurological function 24 h later [63]. Mechanistically, VEGF has been suggested to play a role in the protective effect of AT2R in MCAO. Rats with permanent MCAO treated with an AT2R agonist exhibited faster neurological recovery associated with higher levels of cerebral VEGF compared to vehicle treated permanent MCAO rats. Subsequent experiments suggested AT2R-induced VEGF upregulation involves the mTOR signaling pathway [64]. 

However, not all the actions of AT2R may be beneficial. Emergent evidence suggests that Ang II impacts amyloid precursor protein (APP) metabolism in cerebral microvascular endothelial cells through the Ang II type 2 receptor (AT2R) [65]. The AT2R-mediated action of Ang II on senescent primary human brain microvascular endothelial cells aggravated senescence-induced distortion of APP processing, adding therefore to the development of cerebral amyloid angiopathy and consequently to Alzheimer’s disease pathology [65]. Further experiments are warranted to investigate AT2R involvement as a potential therapeutic target for Alzheimer’s disease management.

The expression of AT2R within the endothelium remains controversial. AT2R endothelial expression appears to be upregulated by endothelial NO. Endothelial cells from porcine aorta, mouse brain, and human umbilical veins subjected to different NO-donor treatments had elevated AT2R mRNA and protein expression through p38 mitogen-activated protein kinase-dependent mechanisms. NO-mediated AT2R upregulation was confirmed in vivo using mice with endothelial-specific eNOS overexpression and others lacking endothelial nitric oxide synthase (eNOS) [66]. However, the specificity of several commercially available AT2R antibodies, one of which is used in confirming endothelial AT2R protein expression, is questionable [67].

In addition to AT1R and AT2R, recent evidence suggests that brain tissue expresses Ang IV receptors (AT4Rs) [68]. Ang IV (or Ang II(3–8), an N-terminal degradation product of Ang II) rescued memory function, enhanced subgranular zone cellular proliferation and dendritic arborization, and lowered cortical and hippocampal reactive oxygen species production in human amyloid precursor protein (APP) transgenic mice. Ang IV also protected neurovascular coupling and endothelial- and smooth muscle cell-induced posterior cerebral artery vasodilation [69]. Those results support the idea of cognitive and cerebrovascular protective effects of AT4R that can counter AngII-AT1R adverse effects. 

### 3.5. Role of Pericytes

Pericytes are multifunctional mural cells located within the basement membrane of microvessels and adjacent to endothelial cells. Pericytes are abundantly present along the walls of brain microvasculature, including pre-capillary arterioles, capillaries, and post-capillary venules, and are a vital component of the neurovascular unit [70]. Studies have reported key roles of pericytes in the regulation of the BBB integrity, modulation of cerebral blood flow, central nervous system scarring, migration of white blood cells through the vascular wall, and promoting angiogenesis and clearance of cell debris [70,71,72]. Central nervous system (CNS) pericytes display multipotent stem cell activity and are affected by the disease state of the body. Some peripheral pathologies of high impact like hypertension, diabetes, kidney diseases, and stroke can impact CNS pericyte morphology, number, and function. Pericyte damage enhances BBB permeability under many pathological conditions, which has been reported to cause neurodegeneration and cognitive impairment [73]. Hypertension is characterized by an increase in mechanical and oxidative stress [74] both of which affect pericyte number [72,75]. In addition, hypertension might decrease pericyte contractility, induce impaired cerebral blood flow autoregulation, pericyte hyperplasia or hypertrophy and disrupt the signals between pericytes and endothelial cells. Similarly, diabetes-induced oxidative stress and amylin production disrupt the tight interaction between pericytes and endothelial cells, cause basement membrane thickening, and trigger pericyte apoptosis. Additionally, diabetes-induced advanced glycation end-product formation enhances fibronectin production in pericytes with associated basement membrane hypertrophy and downregulates claudin-5 expression in microvascular CEC via VEGF and MMP2, thus promoting tight junction damage [76]. Moreover, kidney diseases can increase the rate of calcification and mineralization in pericytes and modify their function [70].

The renin–angiotensin system, a contributor to the pathophysiology of hypertension, diabetes and kidney disease, can alter the function of CNS pericytes [70]. RT-PCR using RNA from human brain pericytes confirmed that both AT1R and AT2R are expressed with a predominant expression of AT1R [71]. Both receptors mediate the response of pericytes to Ang II. Ang II may induce oxidative stress in brain pericytes by increasing the production of Nox4 [71]. In addition, it might trigger pericyte loss and promote leakiness of the blood–brain barrier. Through AT1R, Ang II can increase the contractility of brain pericytes [77]. AT1R also mediates the Ang II-induced influx of calcium to retinal pericytes, and the constriction of the underlying capillaries. The consequent depletion of NO and increase of oxidative stress can also disrupt the endothelium-dependent vasodilation [70]. 

Pericytes do not only interact with endothelial cells via Ang II. Other signals include sphingosine-1-phosphate (S1P), transforming growth factor-beta (TGF-β), and platelet derived growth factor (PDGF), all of which are of upmost importance in the integration of microvessels. TGF-β1 released by pericytes reduces the production of claudin-5, a key component of the tight junctions between adjacent endothelial cells in the blood–brain barrier. In the same context, the interaction between PDGFR-BB and PDGFRβ is crucial for the maintenance of the blood–brain barrier. The absence of either PDGF-BB (released by endothelial cells) or PDGFRβ (the corresponding receptor on pericytes) leads to pericyte loss and blood–brain barrier compromise [70].

## 4. Conclusions and Future Directions

In summary, studies encompassing genetic and pharmacological manipulation of RAS components add to the evidence that targeting RAS may be of clinical significance in slowing or reversing cognitive dysfunctions associated with dementia and Alzheimer’s disease. The observed effects are not only attributed to blood pressure lowering effects of some RAS-targeting drugs, but also to neuro- and vasculoprotective roles including reversing endothelial dysfunction. The involved mechanisms suggest RAS targeting could be effective in different types of dementia and possibly neurodegenerative diseases that comprise similar pathophysiological events.

Investigating the roles of individual ligands and receptors paves the way to pharmacologically target conventional and novel RAS components. The classical ARBs and ACEi are well-tolerated and readily available, but need further randomized clinical trials to confirm their effects on dementia and Alzheimer’s disease. The ACE2/Ang(1–7)/MasR axis constitutes a protective arm of RAS on the blood–brain barrier, but its regulation and interrelationship with classical Ang II signaling is poorly understood. The same is true for the putative cognitive and cerebrovascular protective effects of Ang IV and AT4R. Lastly, a greater understanding of the interface between the brain and systemic RAS is needed, as well as the actions of angiotensins on pericytes that may modulate blood flow and blood–brain barrier function. Investigating these issues should reveal novel drug targets for confronting aging-associated vascular dementia.
