# Correction: Noureddine et al. Impact of the Renin–Angiotensin System on the Endothelium in Vascular Dementia: Unresolved Issues and Future Perspectives. *Int. J. Mol. Sci.* 2020, *21*, 4268

**DOI:** 10.3390/ijms25052995

**Published:** 2024-03-05

**Authors:** Fatima Y. Noureddine, Raffaele Altara, Fan Fan, Andriy Yabluchanskiy, George W. Booz, Fouad A. Zouein

**Affiliations:** 1 Department of Pharmacology and Toxicology, Faculty of Medicine, American University of Beirut, Beirut 1107 2020, Lebanon; nouriddinef@gmail.com; 2 Institute for Experimental Medical Research, Oslo University Hospital and University of Oslo, and KG Jebsen Center for Cardiac Research, 0424 Oslo, Norway; raffaele.altara@medisin.uio.no; 3 Department of Pharmacology and Toxicology, School of Medicine, The University of Mississippi Medical Center, Jackson, MS 39216, USA; ffan@umc.edu (F.F.); gbooz@umc.edu (G.W.B.); 4 Center for Geroscience and Healthy Brain Aging, University of Oklahoma Health Sciences Center, Oklahoma City, OK 73104, USA; andriy.yab@gmail.com

The authors would like to make the following correction to the publication dealing with Reference #3 [1].

We suspect that a typo in one of the drafts caused the wrong citation to be included. Reference 3 should be replaced with this appropriate reference [2].

The authors apologize for any inconvenience caused and state that the scientific conclusions are unaffected. This correction was approved by the Academic Editor. The original publication has also been updated.
